# PET performance evaluation of the small-animal Hyperion II^D^ PET/MRI insert based on the NEMA NU-4 standard

**DOI:** 10.1088/2057-1976/aae6c2

**Published:** 2018-10-24

**Authors:** Patrick Hallen, David Schug, Bjoern Weissler, Pierre Gebhardt, André Salomon, Fabian Kiessling, Volkmar Schulz

**Affiliations:** 1 Department of Physics of Molecular Imaging Systems, Institute for Experimental Molecular Imaging, RWTH Aachen University, Aachen, Germany; 2 Institute for Experimental Molecular Imaging, RWTH Aachen University, Aachen, Germany; 3 Department of Oncology Solutions, Philips Research, Eindhoven, Netherlands; patrick.hallen@pmi.rwth-aachen.de

**Keywords:** positron emission tomography, small-animal imaging, performance evaluation, simultaneous PET/MRI

## Abstract

The Hyperion II^D^ PET insert is the first scanner using fully digital silicon photomultipliers for simultaneous PET/MR imaging of small animals up to rabbit size. In this work, we evaluate the PET performance based on the National Eletrical Manufacturers Association (NEMA) NU 4-2008 standard, whose standardized measurement protocols allow comparison of different small-animal PET scanners. The Hyperion II^D^ small-animal PET/MR insert comprises three rings of 20 detector stacks with pixelated scintillator arrays with a crystal pitch of 1 mm, read out with digital silicon photomultipliers. The scanner has a large ring diameter of 209.6 mm and an axial field of view of 96.7 mm. We evaluated the spatial resolution, energy resolution, time resolution and sensitivity by scanning a ^22^Na point source. The count rates and scatter fractions were measured for a wide range of ^18^F activity inside a mouse-sized scatter phantom. We evaluated the image quality using the mouse-sized image quality phantom specified in the NEMA NU4 standard, filled with ^18^F. Additionally, we verified the *in-vivo* imaging capabilities by performing a simultaneous PET/MRI scan of a mouse injected with ^18^F-FDG. We processed all measurement data with an energy window of 250 keV to 625 keV and a coincidence time window of 2 ns. The filtered-backprojection reconstruction of the point source has a full width at half maximum (FWHM) of 1.7 mm near the isocenter and degrades to 2.5 mm at a radial distance of 50 mm. The scanner’s average energy resolution is 12.7% (Δ*E*/*E* FWHM) and the coincidence resolution time is 609 ps. The peak absolute sensitivity is 4.0% and the true and noise-equivalent count rates reach their peak at an activity of 46 MBq with 483 kcps and 407 kcps, respectively, with a scatter fraction of 13%. The iterative reconstruction of the image quality phantom has a uniformity of 3.7%, and recovery coefficients from 0.29, 0.91 and 0.94 for rod diameters of 1 mm, 3 mm and 5 mm, respectively. After application of scatter and attenuation corrections, the air- and water-filled cold regions have spill-over ratios of 6.3% and 5.4%, respectively. The Hyperion II^D^ PET/MR insert provides state-of-the-art PET performance while enabling simultaneous PET/MRI acquisition of small animals up to rabbit size.

## 1. Introduction

Positron emission tomography (PET) and magnetic resonance imaging (MRI) are two imaging modalities which provide complementary information: PET provides high-sensitivity for *in-vivo* molecular imaging while MRI provides morphological information with high soft-tissue contrast (Judenhofer and Cherry 2013). Additionally, MRI is highly flexible with a multitude of sequences, e.g. offering spectroscopic information, functional MRI (Amaro and Barker 2006) and dynamic data for motion correction (Catana 2015, Chun *et al* 2012).

The integration of simultaneous PET and MRI is challenging: both modalities interfere strongly with each other if not mitigated, and the physical space available for integration of the two modalities is heavily constrained (Vandenberghe and Marsden 2015). To overcome these challenges, we are the first group to develop a simultaneous PET/MR insert with fully digital silicon photomultipliers: the Hyperion II^D^ insert Weissler *et al* 2015. These integrated detectors digitize the signal of each detected photon directly in the photodetector, thus eliminating any interference-prone analog signal transmission over long wires (Timms 1992). This unique approach results in a compact, highly integrated and robust data readout, which facilitates the design of the first small-animal PET/MRI scanner with a wide bore of 200 mm (160 mm with coil) offering imaging of rabbits and other animals of similar size.

The National Electrical Manufacturers Association published standardized protocols for the performance measurement of small-animal PET scanners in the NEMA NU 4-2008 standard (National Electrical Manufacturers Association (NEMA), 2008). The aim of these protocols is to make the performance of different PET scanners comparable in typical imaging conditions. In this work, we present the PET performance evaluation of the Hyperion II^D^ scanner based on NEMA NU-4. Virtually all recent commercial and most research small-animal PET scanners have published performance evaluations based on NEMA NU-4 (Goertzen *et al* 2012, Bao *et al* 2009, Nagy *et al* 2013, Prasad *et al* 2011, Wong *et al* 2012, Szanda *et al* 2011, Cañadas *et al* 2011, Sato *et al* 2016, Gu *et al* 2013).

The comparison to PET performance of other simultaneous PET/MRI scanners is of particular relevance for this work. So far, two other simultaneous small-animal PET/MRI scanners have been evaluated based on the NEMA NU-4 standard (Ko *et al* 2015, Omidvari *et al* 2017)

## 2. Materials and methods

### 2.1. System description

**Figure 1.**
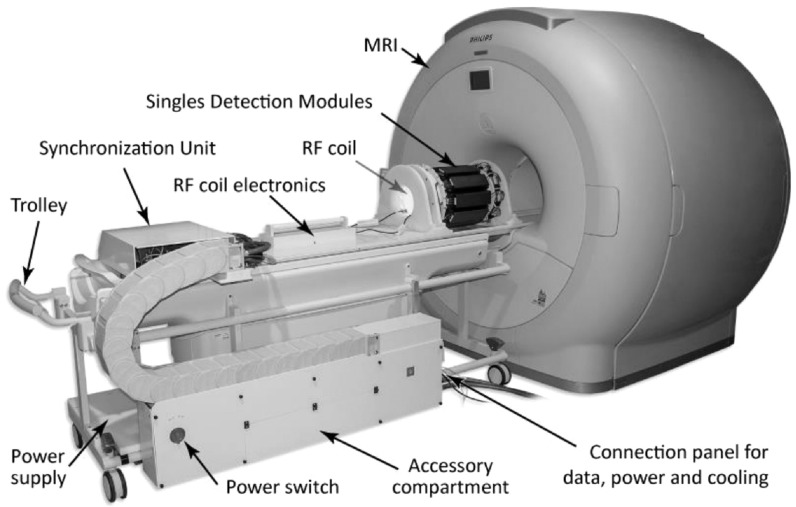
The Hyperion II^D^ PET/MR insert mounted on a patient table in front of a Philips 3-T Achieva MRI system. (Reprinted from (Weissler *et al* 2015); the figure is licensed under a Creative Commons Attribution 3.0 License, see https://creativecommons.org/licenses/by/3.0/).

The Hyperion II^D^ PET/MR insert is a preclinical, MR-compatible PET scanner (Weissler *et al* 2015). The PET gantry is mounted on an MR-compatible trolley and can be moved and operated inside a clinical 3-T Philips Achieva MRI system (Wehner *et al* 2015), as shown in figure 1. We only give a short description of the scanner here, since it has already been described in detail in Weissler *et al* (2015). An initial evaluation of the performance in has been published in Schug *et al* (2016). However, this performance evaluation did not follow the NEMA standard, with the exception of the count rate measurement which was loosely based on the standard, and instead focused on the influence of the scanner’s operation parameters on the performance. Additionally, an earlier version of the data processing was used, using, for instance, a different algorithm for crystal identification (Gross-Weege *et al* 2016). In this work, we chose one set of measurement parameter that we recommend for mouse studies and perform a full NEMA characterization with these settings to allow an objective comparison with the performance of other PET scanners.

The PET ring has an inner diameter of 209.6 mm, measured between the opposing front faces of the scintillation crystals, and an axial field of view of 96.6 mm. This ring consists of 10 singles detection modules, where each of these modules contains 2 × 3 detector stacks. Each detector stack comprises a pixelated lutetium yttrium orthosilicate (LYSO) scintillator array with 30 × 30 pixels with a size of 0.93 × 0.93 × 12 mm^3^ each, placed in a pitch of 1 mm. The scintillator array is glued to an array of digital silicon photomultipliers via a 2 mm-thick glass light guide (Düppenbecker *et al* 2016). The photodetector has an array of 8 × 8 pixels, where each pixel consists of 3200 single-photon avalanche photodiodes (SPADs). This photodetector array has been developed by our group using the digital silicon photomultipliers pixels manufactured by Philips Digital Photon Counting (Frach *et al* 2009). Each SPAD is digitized individually and the computed sum of all SPADs is read out (Frach *et al* 2009). This raw data can either be processed in real-time on the data acquisition and processing server or can be stored for later data processing (Goldschmidt *et al* 2016). The data acquisition server has an individual gigabit ethernet connection to each of the 10 singles detection modules. The gaps between the front faces of the scintillator arrays inside a modules are 3.3 mm in both transversal and transaxial directions and the transversal gaps between the front faces of the scintillator arrays in two neighboring modules are approximately 4 mm.

The MRI transmit/receive coil can be inserted into the PET gantry. In this work, we performed all phantom measurements inside an RF coil dedicated to mouse imaging with an inner bore size of 46 mm. However, an RF coil with a large inner diameters of 160 mm for imaging of larger animals such as New Zealand rabbits is available as well (Weissler *et al* 2015). All point source measurements were performed inside this large rabbit coil to allow the investigation of a larger transversal field of view. The influence of the different RF coils on the PET performance is very small, because CT scans show that they have similar gamma attenuations (Weissler *et al* 2015).

So far, three basically identical systems have been built. The first two systems built have the same components whereas the third system has slightly updated interface electronics, improving the PET performance during extreme MRI gradient switching (Düppenbecker *et al* 2016). However, the measurements in this work have all been performed on the second system with the old interface electronics, which is the same system investigated in Weissler *et al* (2015), Wehner *et al* (2015), Schug *et al* (2016).

### 2.2. Measurements

We performed all measurements using the same settings for the scanner hardware and data processing to make them comparable to each other. These are similar to the settings that we used in Schug *et al* (2016), with the exception of a larger coincidence time window and a maximum-likelihood algorithm for crystal identification.

The used point source is a ^22^Na source with an activity of (0.73 ± 0.07) MBq confined in the center of an acrylic cube with an edge size of 10 mm as specified in the NEMA standard (National Electrical Manufacturers Association (NEMA), 2008). The radionuclide for the two phantom measurements is ^18^F.

Since the assessment of MRI compatibility is not the aim of this work, we performed most measurements outside of the MRI. We’ve previously investigated the MRI compatibility of this scanner conclusively (Wehner *et al* 2015) and can deduce from this work that the results presented here would not differ significantly if taken during simultaneous MRI acquisition. Only the *in-vivo* mouse measurements were performed with simultaneous PET/MRI scans.

#### 2.2.1. Detector settings

The digital photodetector allows the deactivation of the noisiest single-photon cells to reduce the dark noise. We operated the photodetector with the 20% noisiest cells deactivated.

The photodetector has an internal two-level photon trigger. We set the first trigger level to trigger on an average of 3 ± 1.4 photons (trigger setting 3 (Schug *et al* 2016)). After this trigger, the detector enters a 40 ns-long validation phase after which the event is validated and read out if the collected photons satisfy the validation condition. The configured validation condition corresponds to an average of 27.5 ± 10.3 measured photons (validation pattern 0 × 54 in the DPC manual and in other publications (Schug *et al* 2016)). If the detector validates the event, it continues collecting photons for 165 ns.

The system is cooled with a liquid cooling system, which we operated at a temperature of 5 °C resulting in an operating temperature of the photodetectors of 13.7 °C ± 1.4 °C.

#### 2.2.2. Singles and coincidence processing

The measured raw photon values and trigger timestamps were read out and stored for later offline data processing (Schug *et al* 2015). This data processing starts by combining different trigger timestamps and the corresponding photon values of a single gamma interaction to a single cluster. For such a cluster, we selected all timestamps whose timestamps differ less than a cluster time window of 40 ns. Next, we determined the scintillator crystal in which the gamma photon most likely interacted using maximum likelihood estimation with the measured photon counts (Gross-Weege *et al* 2016). The energy was calculated using only the photon counts of the triggered pixels which measured the most photons and its neighboring triggered pixels. We only selected gamma interactions with a measured energy deposition in an energy window of 250 keV to 625 keV. We use this wide energy window to achieve a higher sensitivity and to allow better comparability with other published performance evaluations. When scanning larger objects, such as rabbits, we would recommend a more narrow energy window.

The single gamma interactions were grouped into coincidences using a coincidence time window with a width of 2 ns. The detector’s time resolution would allow the use of a smaller coincidence window to decrease the random fraction at the cost of a slightly decreased sensitivity. For measurements at very high activities we would recommend a smaller coincidence time window, but for most mouse applications the random fraction is already sufficiently small to achieve the best performance using this coincidence window. We only selected coincidences with exactly two triggered detector stacks in the coincidence time window.

We estimated the random count rates ${R}_{{ij}}=2\bar{\tau }\bar{{S}_{i}}\bar{{S}_{j}}$ between to detector elements *i* and *j* using the measured effective singles count rates $\bar{{S}_{i}}$ and the effective coincidence time window $\bar{\tau }$. The effective single count rates $\bar{{S}_{i}}$ and effective coincidence time window $\bar{\tau }$ include corrections based on the coincidence count rate to account for the contribution of true coincidences and pile-up events. (Oliver and Rafecas 2016).

The measurement parameters are summarized in table 1.

**Table 1.** Summary of measurement parameters used in this work.

Measurement parameter	Value
Inhibit threshold	20%
Trigger threshold	3 p.e. (setting 3)
Validation interval	40 ns
Validation threshold	27.5 p.e. (setting 0 × 54)
Integration interval	165 ns
Cluster time window	40 ns
Crystal identification	Maximum likelihood (Gross-Weege *et al* 2016)
Energy window	250 keV to 625 keV
Coincidence time window	2 ns
Cooling temperature	5 °C
Point source activity	0.73 MBq

#### 2.2.3. Spatial resolution

We positioned the ^22^Na point source at different positions in the scanner with increasing radial offsets at two axial positions: the axial center and at one-fourth (i.e. 22.5 mm) of the axial field of view. Our method for the placement of the point source has a precision of approximately 0.3 mm. The axial center of our scanner is exactly between the two central crystal slices, whereas the position at one-fourth of the axial field of view has a slight offset from the center of the corresponding crystal slice. This measurement was performed inside the large rabbit RF coil to allow the investigation of a larger transversal field of view. The duration of the emission scan was 30 min for each source position.

The resulting listmode measurement data were filled into sinograms using the geometrical positions of the crystal detectors into which the crystal identification algorithm placed the gamma interaction. The position inside each 1 × 1 × 12 mm^3^ crystal detector was chosen randomly for each interaction following a uniform distribution for the 1 × 1 mm^2^ front face area and the exponential interaction distribution for the 12 mm length of the crystal. We randomly sampled each coincidence 25 times in this way and filled it in a high-resolution sinogram with a bin size of 0.1 mm for the tangential displacement and an angular bin size of 0.54°. This random sampling is only used for the evaluation of the spatial resolution. We rebin the data into three-dimensional sinograms using single-slice rebinning (Daube-witherspoon and Muehllehner 1987) with a slice thickness of 1mm using only LORs with a ring difference smaller than 4 mm. We reconstructed the resulting sinograms using a 2D filtered backprojection algorithm implemented in the STIR open source reconstruction software (Thielemans *et al* 2012) using an image pixel size of 0.1 mm.

To determine the spatial resolution, we calculated one-dimensional profile lines of the reconstructed point source activity for each dimension by summing up all voxels along the other two dimensions inside a rectangular projection window around the point source. The width of this projection window was twice the determined full width at half maximum (FWHM) of the orthogonal directions. To determine the FWHM, we fit a parabolic function to the three data points with the highest activity to determine the position of the maximum. Then, we linearly interpolated between the data points which lie around the half maximum at both sides of the peak to determine the full width at this position. The full width at tenth maximum (FWTM) was determined analogously. We used the resulting FWHM values to iteratively refine the projection window around the point source. The starting point was the full reconstructed field of view and we used 3 iterations.

#### 2.2.4. Energy resolution and coincidence resolution time

To measure the energy and time resolution, we scanned the ^22^Na source in the scanner’s isocenter for 30 min. We then determined the FWHM of the 511 keV peak in the energy spectrum of the whole scanner from a parabolic fit as described above for the evaluation of spatial resolution. The reported energy resolution Δ*E*/*E* is the FWHM divided by the center of the photopeak. The coincidence resolution time was determined analogously from the spectrum of time differences between the coincident singles.

#### 2.2.5. Scatter fraction, count losses and random coincidences

To measure the count losses, scatter and random rates at high activity, we performed an emission scan of a mouse-sized scatter phantom filled with ^18^F. The scatter phantom was a solid cylinder of high-density polyethylene with a length of 70 mm and a diameter of 25 mm. An off-center hole with a diameter of 3.2 mm diameter was drilled along the axial axis at a radial offset of 17.5 mm to allow the insertion of an activity filled tube. We filled this tube with 0.2 ml water mixed with ^18^F with an activity of 120 MBq.

We placed the filled scatter phantom in the center of the scanner inside the mouse RF coil and performed emission scans every five minutes with increasing scan durations to keep the number of counts per scan approximately constant while the activity decreased.

We filled the measured coincidences into three-dimensional sinograms using single-slice rebinning (Daube-witherspoon and Muehllehner 1987). We applied a cylindrical spatial signal window around the phantom by setting all sinogram bins which are more than 20.5 mm from the sinogram center in each row to zero. This corresponds to only keeping the line of responses which are located less than 8 mm from the edges of the phantom. Subsequently, we shifted each row of the sinogram such that the maximum value in each row is aligned at the center of the sinogram. All total counts were converted to count rates by dividing by the acquisition time.

To determine the combined scatter and random count rates *R*_*S*+*R*_, we first summed up all rates which were located more than 7 mm away from the maximum of each sinogram row. To add an estimate of the random and scatter count rates in the central part of the sinogram, we interpolated linearly between the two bins which are 7 mm from the row maximum. The total coincidence count rate *R*_Total_ is the total sum of all count rates in the sinogram. The true count rate was then determined as ${R}_{\mathrm{True}}={R}_{\mathrm{Total}}-{R}_{S+R}$. The random count rate *R*_*R*_ was estimated during singles processing from the measured singles count rate and the coincidence window (see section 2.2.2). The LYSO scintillators in our scanner have a weak intrinsic radiation because they contain small amounts of the radioactive isotope ^176^Lu. We determined the intrinsic background count rate *R*_Int_ caused by this intrinsic radioactivity from a 30 min long scan without any activity inside the scanner’s field of view. The scatter rate was then determined as the remaining unaccounted count rates:

\begin{eqnarray*}{R}_{S}={R}_{\mathrm{Total}}-{R}_{\mathrm{True}}-{R}_{R}-{R}_{\mathrm{Int}}\end{eqnarray*}

This gives us the scatter fraction as:

\begin{eqnarray*}\mathrm{SF}=\displaystyle \frac{{R}_{S}}{{R}_{S}+{R}_{\mathrm{True}}}\end{eqnarray*}

Additionally, we calculated the noise-equivalent count rate, which is:

\begin{eqnarray*}{R}_{\mathrm{NEC}}=\displaystyle \frac{{R}_{\mathrm{True}}^{2}}{{R}_{\mathrm{Total}}}\end{eqnarray*}

We report all of these count rates in dependence of the average activity concentration during the scans. The activity concentration is the total activity inside the scatter phantom divided by the full volumetric size of the solid scatter phantom, which is $\pi \cdot {12.5}^{2}\,{\mathrm{mm}}^{2}\cdot 70\,\mathrm{mm}\approx 34360\,{\mathrm{mm}}^{3}$. The peak true and noise-equivalent count rates were determined by fitting a quadratic function through the count rate maximum.

#### 2.2.6. Sensitivity

For the measurement of the axial sensitivity profile, we placed the point source in the scanner’s isocenter. We then moved the point source along the axial axis in steps of 1 mm, measuring each axial position for approximately 35 s. We reduced the resulting sinograms of coincidences to three dimensions using single-slice rebinning. We selected only sinogram bins in a signal window of 10 mm around the maximum of each sinogram row to reduce the noise-induced error on the measured sensitivity. We applied the same spatial signal window to the axial dimension by only selecting the nearest 10 sinogram slices around the sinogram slice with highest coincidence count, even though the 10 mm signal window is only specified explicitly for the transversal plane in the NEMA standard. Additionally, we subtracted the measured intrinsic background rate applying the same spatial signal window.

We determined the equivalent sensitivities for mouse and rat applications by averaging the axial sensitivity profile over the representative lengths of 70 mm for mice and 150 mm for rats. Because the scanner’s axial field of view is 96.7 mm, we truncated the sensitivity profile to this length for the equivalent rat sensitivity.

To evaluate the influence of the RF coil, we repeat the measurement of the peak sensitivity in the scanner’s isocenter with the mouse coil and without any coil inside the scanner.

#### 2.2.7. Image quality

The NEMA image quality phantom has three regions: One uniformly filled hot cylinder, 5 hot rods drilled in polyethylene with a diameter from 5 mm to 1 mm and two cold cylinders with an inner diameter of 8 mm and a length of 15 mm in a hot background. The two cold cylinders were filled with air and water respectively. The whole phantom is a cylinder with an inner diameter of 30 mm, length of 50 mm and wall thickness of 1.75 mm.

We filled the hot regions with 20 ml of ^18^F solution and placed the filled phantom in the scanner’s isocenter. The emission scan started at a total phantom activity of 3.7 MBq and lasted for 20 min.

We reconstructed the measurement data with a listmode-based three-dimensional maximum likelihood expectation maximization algorithm including self-normalization and resolution recovery (MLEM-RM) (Salomon *et al* 2012). The reconstruction algorithm grouped the data into 16 ordered subsets with 5 sub-iterations each, regularized with a relative difference prior with dynamic edge preservation parameters (Andreyev *et al* 2017, Nuyts *et al* 2002), and used a voxel pitch of 0.25 mm. Additionally, the reconstruction employs a likelihood-based rejection of inter-crystal detector scatter (Gross-Weege *et al* 2016) and applied corrections for attenuation, scatter and randoms.

To analyze the resulting image quality quantitatively, we placed 8 cylindrical regions of interest (ROI) in the reconstructed activity distribution. The first ROI with a diameter of 22.5 mm and a length of 10 mm was placed in the center of the uniformly filled ROI. We then calculated the mean and standard deviation of the activity in this ROI. The next five ROIs were placed around the hot rods, such that the hot rods were centered in the ROI. Each ROI had a diameter twice the rod diameter and a length of 10 mm. We averaged the voxels along the axial axis and determined the maximum average activity inside each ROI. The recovery coefficient for each rod size is then defined as the ratio of this maximum average and the mean activity in the hot uniform ROI. Additionally, we calculated the standard deviation of each recovery coefficient by calculating the standard deviation of the activity along the axial axis at the position of the maximum average activity. We placed the last two ROIs with a diameter of 4 mm and a length of 7.5 mm inside the center of the two cold cylinders. As a consequence, the spill-over ratios are defined as the ratio of the mean activity inside these ROIs and the mean activity in the hot uniform ROI.

#### 2.2.8. In-vivo mouse measurement

To show the imaging capabilities of our scanner and the settings used for *in-vivo* applications, we scanned a mouse injected with ^18^F-FDG with an activity of 17.1 MBq. The PET scan was started after an uptake time of 1 h with a measurement duration of 20 min. The PET data were processed and reconstructed using the same algorithms and settings as described for the image quality phantom. 16 days before the measurements, human breast tumor cells (MDA-MB-231, 5 ·10^6^ cells in 100 *μ*l PBS) were injected subcutaneously into the hind legs of the mouse.

Simultaneously to the PET scan, we executed a *T*_2_-weighted turbo spin echo (TSE) sequence with a repetition time (TR) of 2400 ms and an echo time (TE) of 100 ms. The sequence had a voxel size of 0.2 mm with an acquisition matrix of 400 × 400 pixels and a slice thickness of 1 mm.

The *in-vivo* mouse measurements were approved by the Maastricht University ethical review committee and were performed according to Dutch national law and the institutional animal care committee guidelines.

Other *in-vivo* measurements of respiratory-and cardiac-gated simultaneous PET/MRI measurement of a mouse heart are shown in Weissler *et al* (2015)

## 3. Results

### 3.1. Spatial resolution

**Figure 2.**
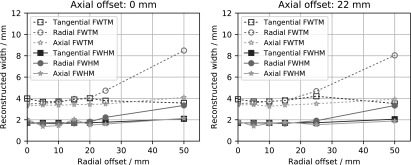
Spatial resolution in dependence of the radial distance of the scanned point source with filtered-backprojection reconstruction. For the left plot, the point source was placed in the scanner’s axial center, and for the right plot, the point source was placed at 1*/*4th of the axial field of view.

Figure 2 shows the spatial resolution for different positions in the scanner’s field of view. The spatial resolution is approximately 1.7 mm full-width at half maximum (FWHM) for radial distances up to 15 mm at both the axial center and 1*/*4th the axial field of view. The full-width at tenth maximum (FWTM) stays mostly below 4 mm for these positions. For larger radial distances, the spatial resolution degrades and a difference in spatial resolution between the axes develops. The cause of this difference is the decagon geometry of the PET scanner where only one axis has orthogonal detectors, as is explained in detail in the discussion section.

### 3.2. Energy and coincidence resolution time

The energy resolution of the whole scanner is 12.73% ± 0.02% (Δ*E*/*E* FWHM) and the coincidence resolution time is (605 ± 1) ps.

### 3.3. Scatter fraction, count losses and random coincidences

**Figure 3.**
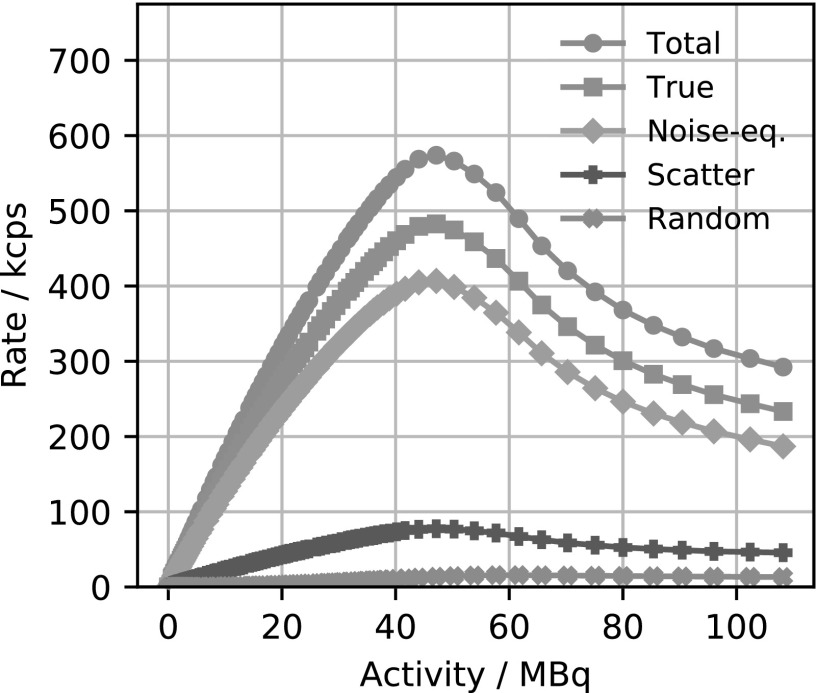
Total, true, noise-equivalent, scatter and random count rates in dependence of the activity concentration in the scatter phantom. The peak true and noise-equivalent count rates are reached at 1.35 kBq*/*mm^3^ with 483 kcps and 407 kcps respectively, which corresponds to a total activity of 46 MBq.

Figure 3 shows the total, true, random, scatter and noise-equivalent count rates in dependence of the activity concentration in the scatter phantom. The true and noise-equivalent count rate peak at an activity concentration of 1.35 kBq*/*mm^3^ with 483 kcps and 407 kcps respectively. This corresponds to a total activity of 46 MBq in the phantom. At this peak, the random rate is 17 kcps, which corresponds to a random fraction of 1.7%. The first derivative of the total count rates stays nearly constant to an activity concentration of approximately 1.2 kBq mm^−3^ and the scatter fraction stays at an approximately constant 13% for activity concentration larger than 0.03 kBq*/*mm^3^. The intrinsic coincidence count rate with no activity inside the scanner is 145 cps.

### 3.4. Sensitivity

**Figure 4.**
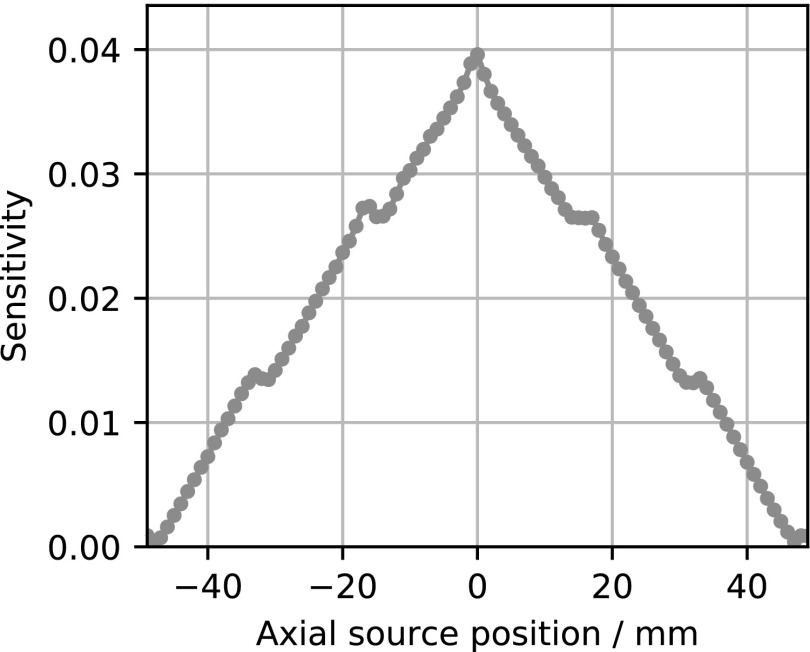
Axial absolute sensitivity profile, the peak sensitivity is 4.0%.

Figure 4 shows the measured absolute sensitivity in dependence of the axial source position. The peak sensitivity is 4.0% ± 0.2%. The average sensitivity for mouse applications is 2.5% ± 0.1% and the average sensitivity for rat applications is 1.9% ± 0.1%. The uncertainties are dominated by the uncertainty on the activity calibration of the point source.

The peak sensitivity is 4.11% ± 0.02% without any coil, 3.95% ± 0.02% with the rabbit coil and 3.93% ± 0.02% with the mouse coil. Here, the given uncertainties are without the uncertainties on the calibration of the activity, since we used the same point source for all measurements and these sensitivity numbers are given to demonstrate the influence of the coils. These given uncertainties are dominated by the positioning uncertainty of the point sources.

### 3.5. Image quality

**Figure 5.**
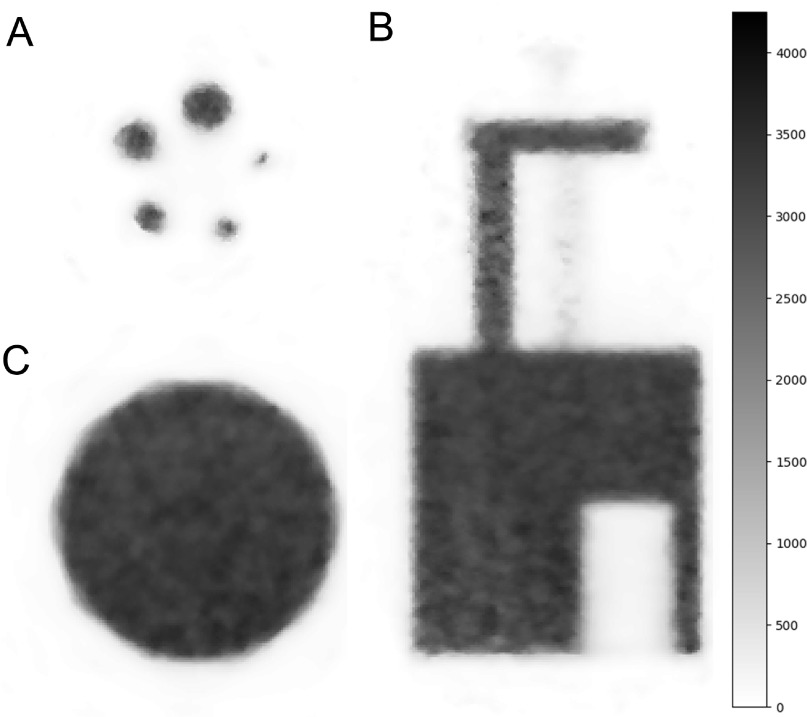
Reconstructed views of the image quality phantom: transverse view of the 5 hot rods (A), coronal view (B), and transverse view of the uniformity region (C). The voxel edge length and slice thickness is 0.25 mm. The images do not have any smoothing or thresholding applied to them.

**Figure 6.**
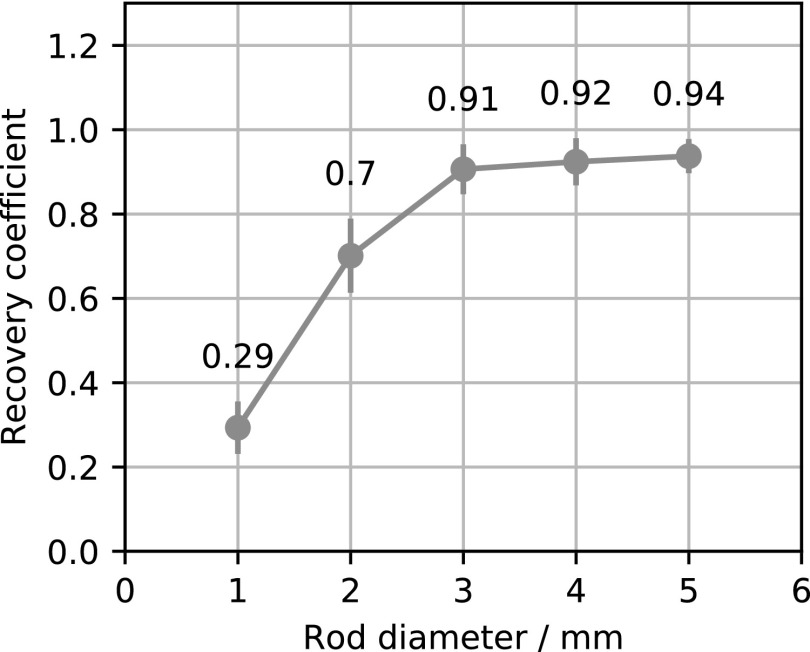
Recovery coefficients of the hot rods with different diameters. The error bars show the standard deviation.

Figure 5 shows views of the reconstructed image quality phantom and figure 6 shows the recovery coefficients for different hot-rod diameters. The mean reconstructed activity in the uniform ROI is 3250 in arbitrary units with a minimum of 2800 and a maximum of 3670. The relative standard deviation of the voxels in the uniformity ROI is 3.7%. The air-filled and water-filled cold ROI have spill-over ratios of 6.3% and 5.4%, respectively, both with relative standard deviations of 14%.

### 3.6. *In-vivo* mouse measurement

**Figure 7.**
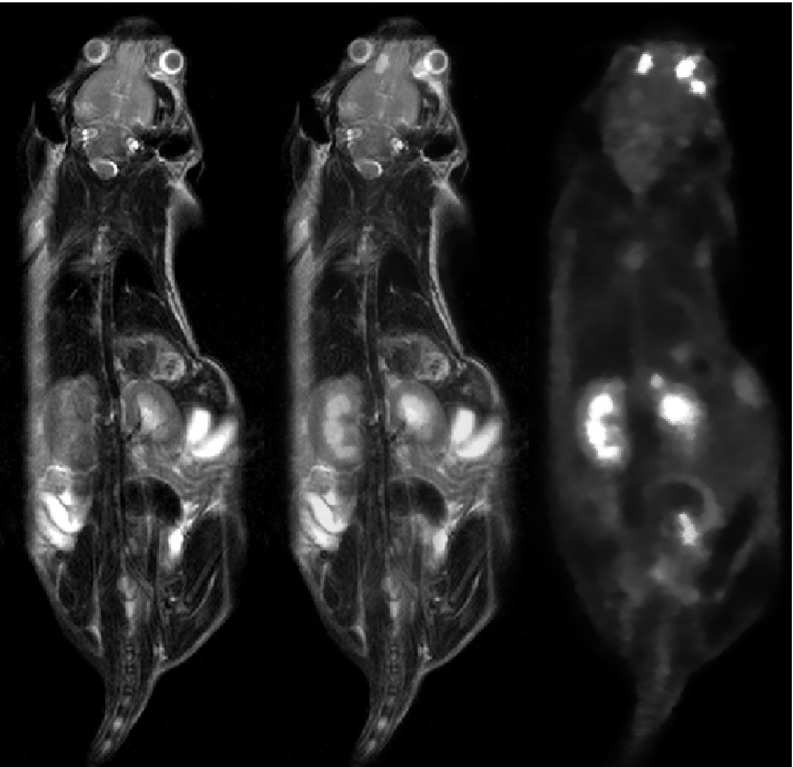
Coronal views of *in-vivo* mouse scans: *T*_2_-weighted MRI scans in greyscale on the left, ^18^F-FDG PET scan in orange on the right, and a fused view of both scans in the center. The accumulation of FDG in the tumor in the right hind leg is visible as a hot spot.

Figure 7 shows the reconstructed results of the simultaneous PET/MRI mouse measurements. The PET image shows high FDG uptake in the kidneys and the Harderian glands behind the eyes and some uptake in the brain, even though the mouse’s head was near the edge of the scanner’s field of view and the uptake and measurement was performed under anesthesia.

## 4. Discussion

### 4.1. Spatial resolution

When using a filtered backprojection reconstruction, the spatial resolution is dominated by star-like artifacts caused by an inhomogeneous detector resolution due to parallax error. These artifacts are a direct consequence of our approach to PET/MRI integration, where we chose a geometry of large, but well-shielded PET modules for minimal interference between PET and MRI. Other PET/MRI systems use either cylindrical shielding enclosing the whole PET ring (Ko *et al* 2015) or do not use any pre-amplifiers and high frequency electronic components inside the MRI, which allows to omit RF shielding altogether (Omidvari *et al* 2017).

Our scanner’s geometry is therefore only an approximation of a ring with a decagon consisting of 10 large modules with a width of 64 mm. This increases the parallax error for line of responses (LOR) which are located farther away from the modules’s center while LORs which hit the modules’s centers are more focused, creating distinct peaks in the sinogram. Simulation studies indicate that such an inhomogeneous detector resolution creates an excess in reconstructed activity along the lines connecting the centers of the modules and the point source, i.e. creating the observed star-like artifact in the data. The transversal gaps of 4 mm between the modules, on the other hand, cause a reduction in reconstructed activity along the lines connecting the gaps and the point source, which in our case does not contribute significantly to the determined spatial resolution. The scanner’s decagon geometry results in a larger parallax error near the center of the field of view compared to more scanners with a more ring-like geometry, for which the parallax error is mostly an issue for activity located farther from the center.

However, all these artifacts are only produced by the filtered backprojection reconstruction, which the NEMA standard mandates and which was consequently used for the evaluation of spatial resolution in this work. With an iterative MLEM reconstruction, which incorporates the scanner’s geometry into the reconstruction, we would be able to achieve a sub-millimeter spatial resolution, as we have previously demonstrated using a hot-rod Derenzo phantom with sub-millimeter structure sizes (Schug *et al* 2016). However, these previously published results use a different set of data processing and reconstruction parameters tuned for optimal spatial resolution. For the results presented here, we used a balanced set of parameters optimized for best image quality and sensitivity.

Despite the use of single-slice rebinning (SSRB), which degrades the axial resolution, the measured axial resolution is close to the two transaxial resolutions, because we drastically reduce the axial blurring by only selecting LORs with a ring difference smaller than 4 mm, i.e. by omitting slanted LORs.

Other PET/MRI inserts achieve a spatial resolution using filtered backprojection of approximately 1.3 mm FWHM at a radial offset of 5 mm (Omidvari *et al* 2017, Ko *et al* 2015, Stortz *et al* 2018). The current state of the art for commercial PET scanners is a reported spatial resolution of down to 1.4mm FWHM (Goertzen *et al* 2012, Bao *et al* 2009, Wong *et al* 2012, Nagy *et al* 2013), which is superior to our reported 1.7 mm.

However, all these scanners have a more ring-like geometry which is advantageous for filtered backprojection reconstruction, and smaller ring diameters which reduces the degrading influence of the gamma’s acollinearity on the spatial resolution. When comparing more realistic iterative reconstructions of Derenzo phantoms, our scanner’s millimeter spatial resolution is close to the current state of the art for PET scanners. This good spatial resolution is mainly achieved through a very small crystal edge size of 0.93 mm, which leads to increased complexity and higher cost as a disadvantage.

### 4.2. Energy resolution and coincidence resolution time

The energy resolution is higher compared to other small-animal PET scanners (Bao *et al* 2009, Szanda *et al* 2011, Ko *et al* 2015, Bergeron *et al* 2009, Gu *et al* 2013), which enables the use of narrow energy windows for scatter reduction, which is especially useful when imaging larger animals such as rabbits.

Unfortunately, the coincidence time resolution is usually not reported in the performance evaluations of small-animals PET scanners, impeding a comparison of our results to other scanners. Ko *et al* (2015) report a coincidence time resolution of 1.33 ns, and the Inveon PET has a reported time resolution of 1.22 ns (Lenox *et al* 2006). Our twice as good coincidence time resolution allows the use of a small coincidence time window of only 2 ns, which leads to the measured small random rates at high activities. When changing the trigger settings of the digital photodetectors to a lower photon threshold, the coincidence resolution time can even be reduced to 250 ps, which enables time-of-flight reconstruction of rabbit-sized objects (Schug *et al* 2016, 2017).

### 4.3. Count rates

The Gigabit Ethernet connections to the singles detection modules are saturated at the activity of the peak coincidence rates. The next bottleneck after the Ethernet connections would be the integration time and ensuing deadtime of the photodetectors, which is approximately 1 *μ*s combined, resulting in a count rate limit a little below of 1000 kcps. The current peak of the count rates at an activity of 46 MBq corresponds to a singles rate of approximately 240 kcps per detector stack, which is well below the count rate limit of the detector. Therefore, future FGPA firmware updates could potentially move the peak of the coincidence rates to higher activities of up to 200 MBq by performing more data processing, such as crystal identification and energy calculation, on the detector stacks instead of transferring the full raw photodetector data to the acquisition server , depending on how fast the FPGA could process the event. However, even the current peak count rates activity of 46 MBq is more than sufficient for most mice applications, which usually inject activities of less than 10 MBq. Applications with rats and rabbits, on the other hand, could profit from a count rate peak at higher activities.

Compared to earlier published results in Schug *et al* (2016), the peak NECR performance is improved by approximately 30%. The cause of this improvement is the maximum-likelihood crystal identification algorithm, which allows the inclusion of events with missing channels (Gross-Weege *et al* 2016) by using the expected photon counts in these channels for interpolation. The center-of-gravity algorithm used in Schug *et al* (2016) would discard such events, leading to decreased sensitivity.

### 4.4. Sensitivity

The axial sensitivity profile shows small deviations from a triangular shape, which are caused by the axial gaps with a size of 3.3 mm between the detector stacks.

A few other small-animal scanners, such as the Inveon PET or the NanoScan sequential PET/MRI, achieve approximately twice the peak sensitivities with 9.32% (Bao *et al* 2009) and 8.4% (Nagy *et al* 2013), while most scanners have similar or smaller peak sensitivities (Goertzen *et al* 2012) using the same energy window. The differences in sensitivity depend primarily on the scanner’s geometries, such as the diameters, axial lengths and scintillator thicknesses. On the one hand, our scanner’s relatively large ring diameter of 209.6 mm reduces the sensitivity, but, on the other hand, enables studies with larger animals such as rabbits, a currently unique feature for simultaneous small-animal PET/MRI scanners.

Because most PET scanner’s have an approximately cylindrical geometry, we can give an analytical model for the expected geometrical contribution to the sensitivity, which consists of three factors. The first factor is the relative solid angle Ω which the cylinder covers:

\begin{eqnarray*}{\rm{\Omega }}=\displaystyle \frac{z}{\sqrt{{z}^{2}+{d}^{2}}}\end{eqnarray*}

with the axial length *z* and the inner diameter *d* of the scanner. The second factor is the fill factor *F*, which includes the gaps between the detector stacks. The fill factor can be approximated as the fraction of scintillator area and cylinder area:

\begin{eqnarray*}F=\displaystyle \frac{\sum {A}_{\mathrm{Scintillators}}}{{A}_{\mathrm{Cylinder}}}=\displaystyle \frac{{n}_{\mathrm{Pixels}}\cdot {x}_{\mathrm{Pixels}}\cdot {y}_{\mathrm{Pixels}}}{\pi \cdot d\cdot z}\end{eqnarray*}

where *n*_Pixels_ is the number of scintillator pixels and *x*_Pixels_ and *y*_Pixels_ are the edge lengths of the scintillator pixel’s front sides. The third factor is the probability *P* that both gamma photons interact with the scintillator:

\begin{eqnarray*}P={(1-{e}^{-{\rm{\Delta }}s\cdot \mu })}^{2}\end{eqnarray*}

where Δ*s* is the scintillator thickness and *μ* is the attenuation length of the scintillator material. For LYSO, the attenuation length at 511 keV is *μ* = 0.084 mm^−1^ (Berger *et al* 2018). As an demonstrative example, the model results in the following geometric sensitivity for our scanner:

\begin{eqnarray*}\begin{array}{rcl}S &amp; = &amp; {\rm{\Omega }}\cdot F\cdot P\\  &amp; = &amp; \displaystyle \frac{96.9\,{\rm{mm}}}{\sqrt{{(96.9{\rm{mm}})}^{2}+{(209.6{\rm{mm}})}^{2}}}\\  &amp;  &amp; \cdot \displaystyle \frac{54000\cdot {(0.93{\rm{mm}})}^{2}}{\pi \cdot 209.6\,{\rm{mm}}\cdot 96.9\,{\rm{mm}}}\cdot {(1-{e}^{-12\cdot 0.084})}^{2}\\  &amp; = &amp; 0.12\end{array}\end{eqnarray*}

Of course, this model is only an approximation of a scanner’s geometric sensitivity. For instance, a significant fraction of LORs will not travel through the full scintillator thickness, which this model does not account for. It also assumes a gamma energy of 511 keV and doesn’t account for scatter.

The ratio between the measured sensitivity and this geometrically maximally achievable sensitivity is *ϵ* = 0.04/0.12 = 0.32 for our scanner. This ratio can be regarded as the detection or processing efficiency. The main contribution to the difference between measured sensitivity and geometric sensitivity is the energy threshold, which, according to Monte Carlo simulations, removes about half of the coincident gamma interactions. Additional effects are losses in the detector and processing chain, due to e.g. not triggered channels, dead time, package loss etc. Thus, this detection efficiency can be used as a benchmark to compare detector and data processing performance of different scanners without the effect of scanner geometry, if the sensitivity was measured using the same energy thresholds.

**Figure 8.**
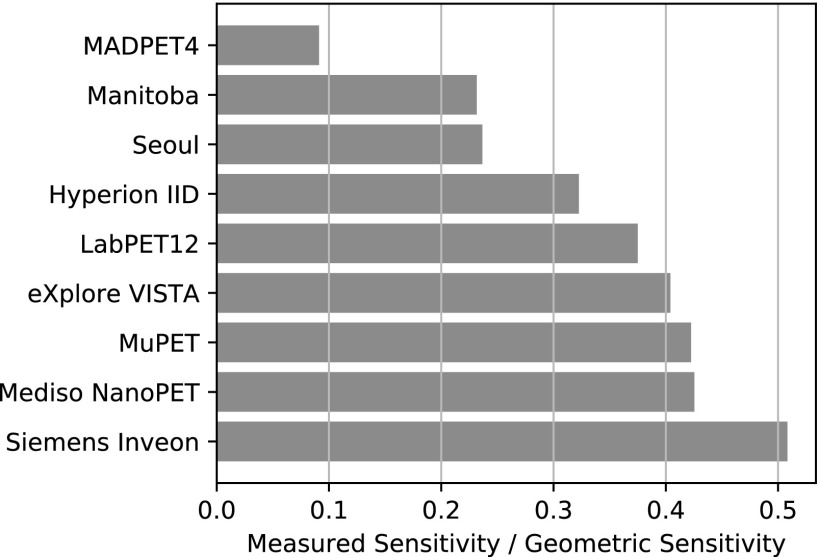
Comparison of ratios between measured and geometric sensitivities for different small-animal PET scanners. All sensitivities were obtained from the following published evaluations based on the NEMA standard with a lower energy threshold of 250 keV: Omidvari *et al* (2017), Stortz *et al* (2018), Ko *et al* (2015), Bergeron *et al* (2009), Wang *et al* (2006), Szanda *et al* (2011), Wong *et al* (2012), Bao *et al* (2009).

Figure 8 shows the ratio between measured and geometric sensitivity obtained from published performance evaluations of small-animal PET scanners. Interestingly, the non-commercial research scanners, i.e. MADPET4, the scanners from the Universities of Manitoba and Seoul, and our scanner, have the worst ratios. Additionally, these are also the only simultaneous PET/MRI scanners in the list, so one contributing factor to these smaller ratios might be the attenuation by the RF coils. Our scanner achieves a ratio of *ϵ* = 0.32, which is the best of the simultaneous PET/MRI scanners and about 30% below the ratios of the dedicated commercial PET scanners. The ratios of these scanners range from 0.39 to 0.51, with the Inveon PET achieving the highest ratio.

### 4.5. Image quality

Overall, the quantitative results of the image quality phantom measurement match the state of the art of small-animal PET scanners. The achieved uniformity of 3.7% is better than all other published scanners (Bao *et al* 2009, Kim *et al* 2007, Prasad *et al* 2011, Goertzen *et al* 2012) with the exception of the NanoScan PET/MRI, which achieves a uniformity of 3.5% (Nagy *et al* 2013). However, these results are strongly affected by the sensitivity and the image reconstruction. The Inveon PET (Bao *et al* 2009), the microPET Focus 120 scanner (Kim *et al* 2007) have a higher sensitivity with a worse uniformity, while the MADPET4 (Omidvari *et al* 2017) achieves a 8% uniformity with a much smaller sensitivity of 0.72%. The recovery coefficients are either better or similar to most other scanners, depending on the rod diameter: For the smallest diameter of 1mm we achieve the best recovery with 0.29, while for the other rods the NanoScan PET/MRI, the MADPET4 and the LabPET (Bergeron *et al* 2009) have slightly higher recovery coefficients. Simulation studies indicate that our recovery performance is mainly limited by inter-crystal detector scatter, which is not included in the resolution recovery of our image reconstruction. Collimator measurements of our detector stack have shown that the crystal identification in our light-sharing detectors performs well for non-scattered events (Ritzer *et al* 2017). Additionally, the recovery performance improves when using higher energy thresholds at the price of degrading uniformity.

The spill-over ratios of approximately 6.3% and 5.4% for the two cold regions are similar to most other PET scanners that also apply scatter and attenuation corrections. Examples of scanners that achieve smaller spill-over ratios in at least one of the regions are Inveon (Bao *et al* 2009), Argus (Wang *et al* 2006), and microPET Focus 220 (Goertzen *et al* 2012). However, most small-animal PET scanners do not apply attenuation and scatter corrections in their performance evaluations, unsurprisingly resulting in substantially larger spill-over ratios (Omidvari *et al* 2017, Ko *et al* 2015, Nagy *et al* 2013, Prasad *et al* 2011, Gu *et al* 2013).

## 5. Conclusion

The presented work is a full PET performance evaluation based on the NEMA NU-4 protocol of a small-animal simultaneous PET/MRI system. Despite the challenging nature of PET/MRI integration, which requires trade-offs in the system design, the overall PET performance is comparable to state-of-the-art commercial small-animal PET scanners and the sensitivity exceeds the sensitivity of other simultaneous small-animal PET/MRI systems due to the larger axial field of view.

Additionally, our scanner proves the viability of digital silicon photomultipliers for simultaneous PET/MR imaging for the first time, achieving a robust and compact integration of PET detector electronics.
